# Electrical synapses interconnecting axons revealed in the optic nerve head – a novel model of gap junctions’ involvement in optic nerve function

**DOI:** 10.1111/aos.14272

**Published:** 2019-10-10

**Authors:** Adrian Smedowski, Saeed Akhtar, Xiaonan Liu, Marita Pietrucha‐Dutczak, Lucia Podracka, Elisa Toropainen, Aljoharah Alkanaan, Marika Ruponen, Arto Urtti, Markku Varjosalo, Kai Kaarniranta, Joanna Lewin‐Kowalik

**Affiliations:** ^1^ Department of Physiology School of Medicine in Katowice Medical University of Silesia Katowice Poland; ^2^ Department of Optometry College of Applied Medical Sciences King Saud University Riyadh Kingdom of Saudi Arabia; ^3^ Institute of Biotechnology University of Helsinki Helsinki Finland; ^4^ School of Pharmacy University of Eastern Finland Kuopio Finland; ^5^ Department of Ophthalmology University of Eastern Finland Kuopio Finland; ^6^ Department of Ophthalmology Kuopio University Hospital Kuopio Finland

**Keywords:** conduction resistance, electrical synapses, impulse conduction, optic nerve, retinal ganglion cell axons

## Abstract

**Purpose:**

To characterize newly discovered electrical synapses, formed by connexin (Cx) 36 and 45, between neighbouring axons within the optic nerve head.

**Methods:**

Twenty‐five Wistar rats were killed by CO_2_ inhalation. Proximal and distal optic nerve (ON) stumps were collected and processed for immunostainings, electron microscopy (EM) with immunogold labelling, PCR and Western blots (WB). Additional 15 animals were deeply anaesthetized, and flash visual evoked potentials (fVEP) after retrobulbar injection of saline (negative control) or 100 *μ* m meclofenamic acid solution (gap junctions’ blocker) were recorded. Human paraffin cross‐sections of eyeballs for immunostainings were obtained from the Human Eye Biobank for Research.

**Results:**

Immunostainings of both rat and human ON revealed the presence of Cx45 and 36 colocalizing with *β*3‐tubulin, but not with glial fibrillary acidic protein (GFAP). In WB, Cx36 content in optic nerve was approximately halved when compared with retina (0.58 ± 0.005 in proximal stump and 0.44 ± 0.02 in distal stump), Cx45 showed higher levels (0.68 ± 0.01 in proximal stump and 0.9 ± 0.07 in distal stump). In immunogold‐EM of optic nerve sections, we found electric synapses (formed mostly by Cx45) directly coupling neighbouring axons. In fVEP, blocking of gap junctions with meclofenamic acid resulted in significant prolongation of the latency of P1 wave up to 160% after 30 min (p < 0.001).

**Conclusions:**

Optic nerve (ON) axons are equipped with electrical synapses composed of neuronal connexins, especially Cx45, creating direct morphological and functional connections between each other. This finding could have substantial implications for understanding of the pathogenesis of various optic neuropathies and identifies a new potential target for a therapeutic approach.

## Introduction

The biology of connexins, proteins that form gap junctions (GJs), is relatively well described. It is known that in situations of cell stress, connexins may undergo misfolding and, as protein waste, are degraded in proteasomal pathways (i.e., lysosomes and autophagy) (Orellana et al. 2013; Su & Lau 2014). Transient passage of ions and small molecules, such as glutamate, glutathione, ADP and glucose, must have certain impacts in the cells that can be both beneficial and harmful. Gap junctions (GJs) have been described to be involved in both pro‐survival and pro‐death activity (Carette et al. 2014). An excess of glutamate is considered to be the main cause of excitotoxicity (Gauthier & Liu 2016). It is hypothesized that passage of glutamate through GJs can mediate spreading of excitotoxic insults between neurons, resulting in programmed cell death; however, the role of GJs in apoptosis has not yet been fully determined (Takeuchi et al. 2008; Akopian et al. 2014, 2017). On the other hand, GJs allow for passage of energetic substrates and regulatory molecules (e.g., glutathione) between neurons, participating in cell rescue processes (Abrams & Rash 2009). The involvement of glial electrical synapses in the pathology of optic neuropathies has been suggested before (Kerr et al. 2010; Chen et al. 2015). A decrease in GJ density in astrocytes, due to high hydrostatic pressure, is postulated to be involved in the pathomechanism of glaucomatous neurodegeneration (Malone et al. 2007). Gap junctions (GJs) between excitable cells buffer the intracellular environment and form an electrical web, creating alternative pathways for conduction of impulses (Maxeiner 2005; Abrams & Rash 2009). It is known that GJs’ conductivity can modulate signal propagation in the retina (Maxeiner 2005). Dopamine released as a result of light stimulation in horizontal, and amacrine cells activates protein kinase A, which phosphorylates connexin 36 (Cx36) and leads to reduced conductance of electrical synapses composed by this protein (Urschel et al. 2006). In the optic nerve (ON), it has been shown previously that GJs formed by connexin 43 (Cx43) couple astrocytes with neighbouring axons, but axons themselves form independent pathways that conduct action potentials towards the brain with no collateral impulse spreading (Quigley 1977). This arrangement would make the impulse conduction in the ON very fragile since any disturbance within a single axon might result in the blockage of action potential conduction.

Here, we show for the first time that ON axons are equipped with electrical synapses composed of neuronal connexins, especially connexin 45 (Cx45), creating direct morphological and functional connections between each other. These newly described details regarding ON structure provide new insight into its conductivity properties, since the described GJs may modulate ON electrical resistance.

## Materials and methods

### Animals

Experimental procedures involving animals were approved by the Finnish National Animal Ethics Committee in the State Provincial Office of Southern Finland and the Local Committee for Animal Experiments of Medical University of Silesia in accordance with the ARVO Statement for the Use of Animals in Ophthalmic and Vision Research and the EC Directive 86/609/EEC for animal experiments. Animals were provided by the Laboratory Animal Center, University of Eastern Finland, Kuopio, Finland and by the Center for Experimental Medicine, Medical University of Silesia, Katowice, Poland. For the purpose of the study, we used forty‐male Wistar rats weighing 160–180 g. Fifteen animals were utilized for functional recordings, for which general anaesthesia with an intraperitoneal injection of a mixture of ketamine (50 mg/kg; Ketalar, Pfizer Oy Animal Health, Finland) and medetomidine (0.4 mg/kg; Domitor, Orion Oy, Finland) was applied. Other animals were used for protein and gene expression analysis and imaging, which included Western blots (*n* = 4 animals), PCR (*n* = 4 animals), immunostainings (*n* = 7 animals) and electron microscopy (*n* = 10 animals). Animals were killed by CO⁠_2_ inhalation and subsequent decapitation; eyes with approximately 6‐mm stump of optic nerve were enucleated and processed for further analyses.

### Human samples

Human paraffin longitudinal sections of eyes with optic nerves (*n* = 2) were received from the Human Eye Biobank for Research, St. Michael's Hospital, University of Toronto, Canada, under permission from the local ethical committee. The paraffin sections, after deparaffinization in xylene and rehydration in a decreasing gradient of alcohol, underwent standard immunostaining procedures.

### Western blots

For Western blotting, retinas and optic nerves were lysed with T‐PER™ Tissue Protein Extraction Reagent (Thermo Fisher Scientific, Waltham, MA, USA) supplemented with Sigma FASTTM Protease Inhibitor Coctail (Sigma‐Aldrich, Saint Louis, MO, USA), according to the manufacturers’ protocols. Protein concentrations were measured using Bradford reagent (Sigma‐Aldrich), and 10 *μ*g of total protein extracts were used for 10% SDS‐PAGE. After transfer, nitrocellulose membranes (Amersham, UK) were blocked and incubated with the following dilutions of primary antibodies in 3% BSA: connexin 36 (dilution: 1:1000, sc398063), connexin 45 (dilution: 1:1000, ab70365), *α*‐tubulin (dilution 1:1000, mca77p), according to the manufacturers’ protocols. Horseradish peroxidase (HRP)‐linked secondary antibodies were used, and signals were visualized by chemiluminescence using the Amersham ECL Western Blotting analysis system (GE Healthcare). The band intensities of blots were quantified using ImageJ software (http://imagej.nih.gov/ij/).

### PCR analysis

Rat optic nerve stumps and retinas were preserved in RNAlater (Qiagen, Netherlands) immediately after eye enucleation. After RNA extraction using an RNAeasy mini kit (Qiagen) and DNAse (DNA free, Ambion, Austin, TX, USA) treatment of the samples, cDNA was synthesized by reverse transcription using M‐MuLV (Fermentas, Hanover, MD, USA). SYBR Green Master Mix (Applied Biosystems) and primers, Cx45 (NM_001085381.1 Rattus norvegicus gap junction protein, gamma 1), forward 5′‐TGGCTCACTGTGCTGATTGT‐3′, reverse 5′‐CTGGAATACCCAGAAGCGCA‐3′ (Oligomer, Helsinki, Finland); commercially available Gjd2 (NM_019281 Rattus norvegicus gap junction protein, delta 2; cat. n. 330001‐ PR444550A, Qiagen), were used for cDNA amplification and detection in an ABI Prism 7500 (Applied Biosystems). The qPCR‐amplified products were verified by DNA polyacrylamide gel electrophoresis where 8.3 *μ*l of qPCR product was mixed with 1.7 *μ*l of 6× Loading dye (Thermo Fisher Scientific) and added to the wells of a 4%–20% MINI‐Protean TGX Precast gel (BioRad, Hercules, CA, US). 5 *μ*l of GeneRuler Ultra Low Range DNA Ladder (SM1213, Thermo Fisher Scientific) was added to the wells flanking the row of samples. The gel was run for 24 min at 200 V in TBE buffer. After the run, the gel was poststained in GelGreen (Biotium) for 30 min according to manufacturer's instructions and then imaged using the Bio‐Rad Gel Doc 2000 system.

### Immunofluorescence staining

Rat retinas were isolated from eyeballs, mounted on microscope slides and postfixed overnight in 4% paraformaldehyde (PFA) at +4°C. The next day, retinas were washed 3 times in 0.1 M TBS and blocked for 30 min in 10% Normal Goat Serum (NGS)/0.1 M TBS/0.1% Triton solution. Floating retinal samples were then incubated overnight at +4°C with the following antibodies: connexin 36 (dilution: 1:100, sc398063), connexin 45 (dilution: 1:100, ab70365), *β*3tubulin (tuij1, dilution 1:300, ab17207) and GFAP (dilution 1:500, G3893). Secondary antibodies were applied for 3 hr at room temperature (RT) after tissue samples were washed three times in 0.1 M TBS. For secondary antibodies, we used AlexaFluor 488 or 594 (Thermo, dilution 1:500). Retina samples after final washing were mounted with Mowiol containing DAPI (dilution 1:10 000). Rat optic nerve longitudinal paraffin sections (5 *μ*m thick) and human eyeball cross‐sections underwent a staining procedure similar to that of the whole‐mounted retinas.

### Electron microscopy

To study the ultrastructure of gap junctions, intermediate filaments, neurofilaments and microtubules of axons in the optic nerve head, healthy rat eyes were processed for electron microscopy. Optic nerves were fixed in 2.5% glutaraldehyde in 0.1 M PBS immediately after the death of the animal. The tissue was washed with 0.1 M PBS (3 × 15 min), postfixed in osmium tetroxide for one hour, then washed with water (3 × 15 min) and dehydrated through a graded ethanol series (50%–100%) 15 min each and 100% acetone in the end (2 × 30 min). Optic nerve stumps were infiltrated with a mixture of acetone and Spurr resin (1:1 and 1:2) for 2 hr each. The tissue was further infiltrated with 100% Spurr resin (3 × 8 hr) and embedded in Spurr resin for 8 hr at +70°C. Semithin and ultrathin sections were cut from blocks using an RMC ultra‐cut microtome. All the sections were cut at a 180‐degree angle parallel to the longitudinally running optic nerve fibres (tangential section) through the middle of the optic nerve head to get sections of longitudinally running axons and astrocyte processes. Semithin (1 *μ*m) sections were collected on glass slides and stained with toluidine blue. The ultrathin sections were collected on 200 mesh copper grids. The sections were stained with 2% uranyl acetate (10 min) and lead citrate (10 min) and observed using a Jeol 1400 transmission electron microscopy (Jeol Ltd, Akishima, Japan). The digital images were taken by using the iTEM programme. The images were captured by a bottom‐mounted Quemesa camera.

The differentiation between axons and astrocytes was based on the presence and diameter of intracellular actin filaments and microtubules (Table 1) (Peters & Vaughn 1967).

**Table 1:** Diameter of microtubules and neurofilaments in axons, and intermediate filaments (microfilaments) in astrocytes

Structure	Mean diameter (nm) ± SD *n* = 17
Microtubules within axons	25.70 ± 5.93
Neurofilaments within axons	12.18 ± 1.75
Intermediate filaments within astrocytes	11.67 ± 5.21

The differentiation between axons and astrocytes was based on the presence of microtubules and neurofilaments within axons and intermediate filaments within astrocytes. Measurements were performed on ultrathin longitudinal sections of rat optic nerves using electron microscope imaging.

### Immunogold labelling

Optic nerve heads were fixed in 4% paraformaldehyde (PFA) with 0.5% glutaraldehyde in 0.1M PBS for 2 hr at +4°C within 30 min after removal from the eye. The tissue was embedded and polymerized in LR White (Akhtar et al. 2001). Ultrathin sections were cut from the blocks using an RMC Ultra‐cut Microtome and collected on 200 mesh formvar/carbon nickel grids. Rabbit polyclonal antibody Cx45 (dilution 1:100) was used as the primary antibody to localize connexin in the gap junctions in the optic nerve head. The antibody was then labelled with 10 nm goat anti‐rabbit immunogold conjugate (1:25). The immunogold conjugates, goat serum, bovine serum and Tween‐20 were supplied by Biocell, Cardiff, UK. Immunolabelling of the sections was performed as described previously (Akhtar et al. 2001). Sections were stained with 2% aqueous uranyl acetate and lead citrate and analysed using a JEOL 1400 transmission electron microscope.

### Visual evoked potentials

Animals were deeply anaesthetized, the skin on the head was dissected to visualize skull structures, and bleeding vessels were coagulated with 70% ethanol. Three holes were drilled using a 1 mm diameter drill (Fig. S1). Stainless steel screws (plain end, pan head, Philips drive, 3/6″ length, #0–80 threads) were placed in holes as electrodes. Wires were connected with 3 screws, 2 active and one reference electrode, and a tail needle electrode was used as a ground. Pupils were dilated with 1% Tropicamide and protective Viscotears were placed to prevent corneal desiccation. Recordings were performed using the Color Dome system (Diagnosys, Lowell, MA, USA). Animals were kept under red light for 5 min, then a series of five recordings followed by a 1 min break under red light were performed with the following parameters: bright light flash 3 (P)cd/m^2^, 6500K, frequency 1 Hz, time of measurement 200 ms, sample rate 5 kHz. Responses were amplified 20 000 times with low and high band‐pass filter settings of 1 and 100 Hz. Depending on the study group, animals received retrobulbar injection of saline (50 *μ*l, *n* = 5) or retrobulbar injection of 100 *μ* m meclofenamic acid (50 *μ*l, *n* = 5). Reference animals received no injections. Measurements were repeated at time‐point 0, 30, and 60 min, where 0 means the time‐point before retrobulbar injection (basic recording), and 30 and 60 min referred to time after retrobulbar injection was done. Animals were killed by CO_2_ inhalation when the last recording had been completed.

### Statistics

IBM SPSS (Armonk, NY, USA) was used for the statistic evaluation. Descriptive statistics are shown as the mean ± standard deviation (SD). Multiple comparisons between groups were performed using related samples Friedman's analysis and comparisons between two groups were performed using paired‐samples Wilcoxon test. p values < 0.05 were considered statistically significant.

## Results

### The presence of Cx36 and Cx45 transcript and protein in retina and ON has been confirmed in molecular analyses

**Figure 1:**
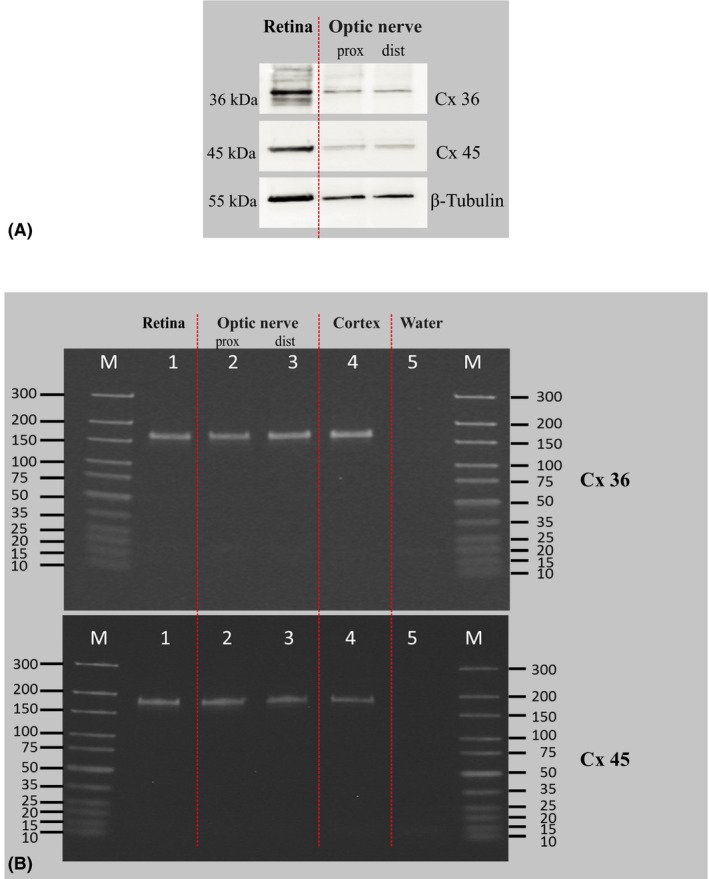
Molecular analysis of Cx 36 and Cx 45 transcript and protein in retina and ON homogenates. (A) representative Western blot of homogenates prepared from rat retina and proximal and distal optic nerve stumps to detect the presence of Cx36 and Cx45. For the WB analysis, 2 retinas or 2 optic nerve stumps were pooled to obtain proper protein content. ON = optic nerve. (B) representative PCR of homogenates prepared from rat retina and proximal and distal optic nerve stumps to detect the presence of Cx36 and Cx45 transcripts. For the PCR analysis, 2 retinas or 2 optic nerve stumps were pooled to obtain proper RNA concentration. Rat brain cortex was used as a positive control and RNA‐free water as a negative control. ON = optic nerve.

We analysed homogenates of uninjured rat retina and proximal and distal stumps of the ON (i.e., unmyelinated and myelinated regions of the ON) using Western blot (WB). We found that Cx36 content in the ON was approximately two folds lower than in the retina (0.58 ± 0.005 in the proximal stump and 0.44 ± 0.02 in the distal stump, for the retinal level = 1.0), while Cx45 was present at higher levels in the ON compared to Cx36 (0.68 ± 0.01 in the proximal stump and 0.9 ± 0.07 in the distal stump, for the retinal level = 1.0) (Fig. 1A). Additionally, we detected the presence of Cx36 and Cx45 transcript via PCR analysis of the retina and the proximal and distal ON homogenates (Fig. 1B).

### Gap junction markers Cx36 and Cx45 are colocalizing with RGCs’ Tuij1 protein in immunostainings

**Figure 2:**
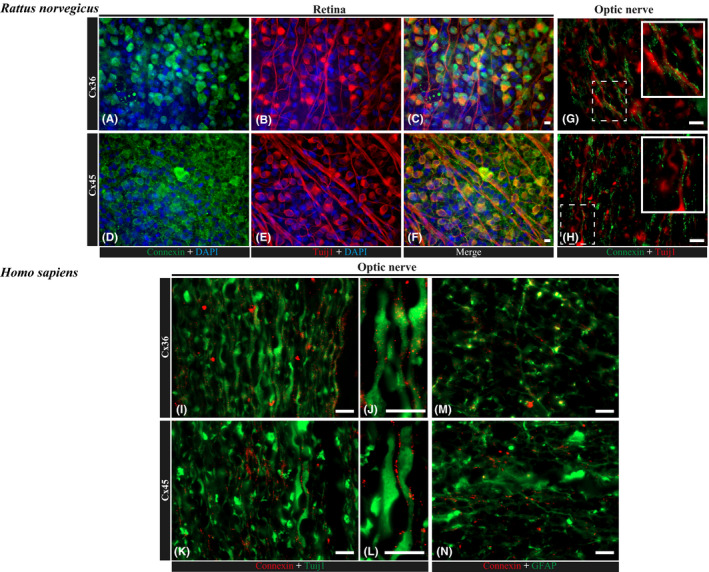
Expression and localization of connexin 36 and 45 in rat and human retina and optic nerve. (A–F) immunofluorescence staining of rat, whole‐mounted retinas for Cx36 (A–C) and Cx45 (D–F) in colocalization with RGC marker, Tuij1 (*β*3tubulin); nuclei are counterstained with DAPI. Scale bar, 10 *μ*m. In these whole‐mounted retina photographs, Cx36 expression within RGCs was present mostly intracellularly, while Cx45 showed a cell membrane pattern. In ON longitudinal sections, staining for both markers (Cx36 and Cx45) was observed along Tuij1‐positive axons. (G, H) immunofluorescence staining of paraffin longitudinal sections of a rat optic nerve head region for Cx36 (G) and Cx45 (H) in colocalization with RGC marker, Tuij1 (*β*3tubulin). Scale bar, 10 *μ*m. (I–N) immunofluorescence staining of longitudinal paraffin sections of a human optic nerve head region for Cx36 (I, J) and Cx45 (K, L) in colocalization with RGC marker, Tuij1 (*β*3tubulin), and Cx36 (N) and Cx45 (M) in colocalization with glial cell marker, GFAP. Scale bars, 10 *μ*m (I, K, N, M); 2 *μ*m (J, L).

**Figure 3:**
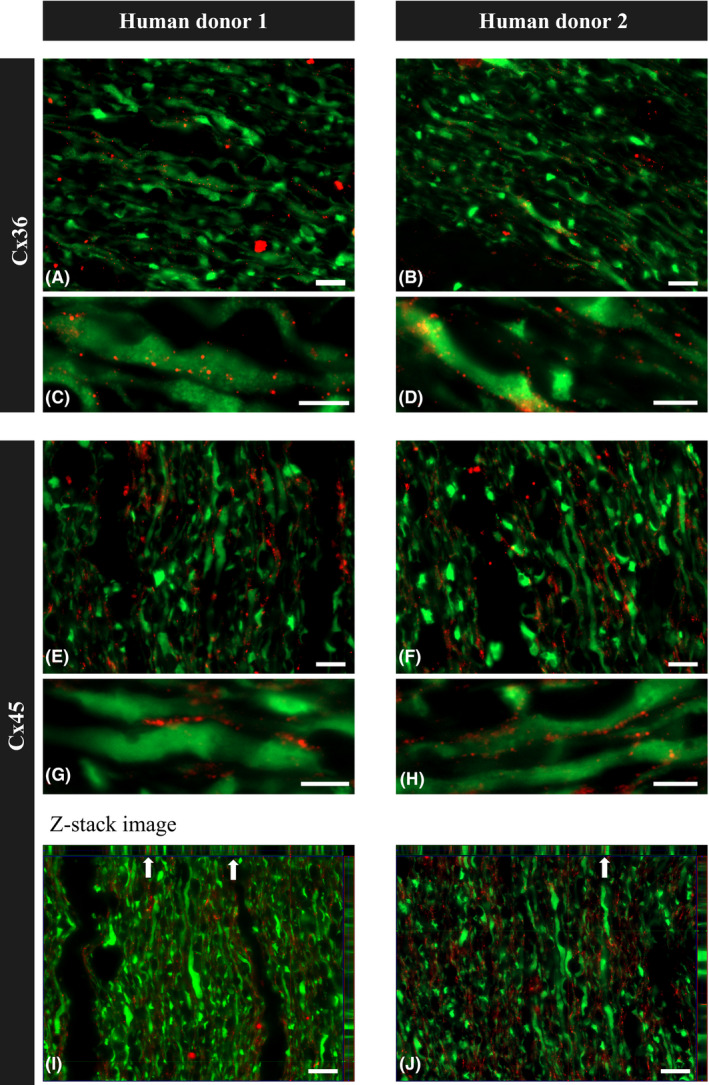
Immunofluorescence staining of paraffin longitudinal sections of the ON from two healthy human donors. (A–D) immunostaining for Cx36 and Tuij1 showed dots of Cx36 staining colocalized with Tuij1‐positive ON axons. (E–H) immunostaining for Cx45 and Tuij1 showed dots of Cx45 staining colocalized with Tuij1‐positive ON axons. (I, J) photographs show Z‐stack imaging of longitudinal paraffin sections of the human optic nerve head region stained for Cx45 and Tuij1, with arrows pointing to the colocalization of axons with Cx45 projected in two planes. Scale bars, 10 *μ*m (A, B, E, F, I, J); 5 *μ*m (C, D, G, H).

Because tissue homogenate analysis did not give us information about connexins localization, we performed immunostainings on rat whole‐mounted retinas, longitudinal sections of the rat and human ON for Cx36 and Cx45 and colocalization with RGCs’ marker, *β*3tubulin (Tuij1), or glial cell marker, glial fibrillary acidic protein (GFAP). In these stainings, different patterns for Cx36 and Cx45 were observed in whole‐mounted rat retinas, where Cx36 was localized intracellularly within RGCs and Cx45 formed dots on cell surfaces (Fig. 2A–F). In the ON, Cx45 was clearly visible as dots present between neighbouring axons, which were positive for *β*3tubulin, and did not colocalize with glial cells stained with GFAP (Fig. 2M,N). Cx36 showed more subtle staining, confirming the WB finding that Cx45 is the dominant neuronal gap junction protein in the ON (Fig. 2G–L). Example analysis of immunostaining of human ON longitudinal sections from two human donors is presented on Fig. 3. It is important to highlight that we observed similar patterns of ON staining in both rat and human tissue.

### Electron microscopy revealed three types of gap junctions within ON structure

**Figure 4:**
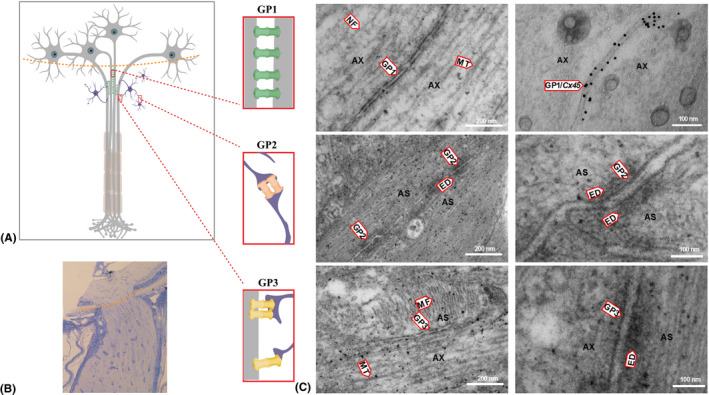
Electron micrographs (EM) of longitudinal sections of a rat optic nerve head (ONH). (A) schematic picture depicting interconnections of axons and astrocytes within the ONH. There are three types of gap junctions identified in the ONH: gap junctions between two axons (GP1), between two astrocytes (GP2) and between axons and astrocytes (GP3). (B) light micrograph of a longitudinal section of rat optic nerve with marked level of ONH (red dashed line). (C) EM presenting different types of synaptic interconnections within the ONH. Upper panel shows fragments of two axons (AX) containing microtubules (MT), neurofilaments (NF) and gap junctions between two axons (GP1); the right EM shows GP1 with immunogold labelling of Cx45 within the gap junctions between two axons (GP1/Cx45). Middle panel shows an EM of fragments of two astrocytes (AS) showing gap junctions (GP2) between them. GP2 contains a double membrane and electron‐dense material (ED) around the electric synapse. The lower panel shows an EM of a fragment of an astrocyte (AS) and axon (AX) with a gap junction between them (GP3), microtubules (MT) in the axon, actin filaments (MF) in the astrocyte and electron‐dense material (ED) on the astrocytic side of GP3.

**Figure 5:**
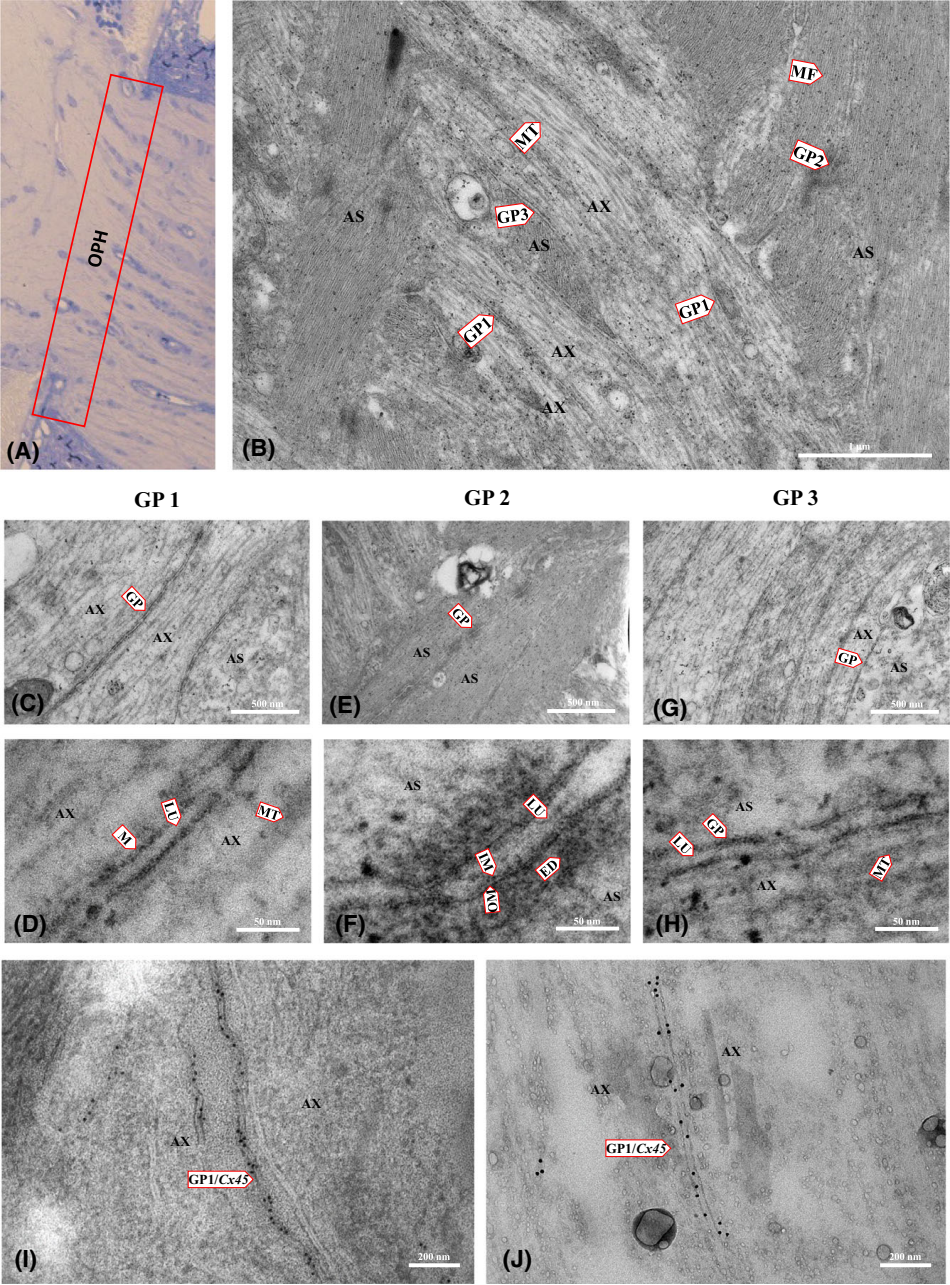
Electron micrographs (EM) showing the ultrastructure of different interconnections within the rat optic nerve head. (A) light microscopy of the optic nerve head showing the region of EM imaging. (B) EM of a longitudinal ultrathin section of a rat optic nerve head showing the ultrastructure of cellular components: microtubules (MT) of axons (AX), microfilaments (MF) of astrocytes (AS) and gap junctions between two axons (GP1), between two astrocytes (GP2), and between axons and astrocytes (GP3). (C, D) EM showing a fragment of two axons with microtubule (MT) and GP1 details: lumen (LU), membrane (M). (E, F) EM of a fragment of two astrocytes and GP2 showing lumen (LU), inner membrane (IM), outer membrane (OM) and electron‐dense material (ED) around the gap junction. (G, H) EM of a fragment of the astrocyte and axon showing GP3 between them and microtubules in the axon. (I, J) immunogold labelling for Cx45; the EM shows a fragment of two axons with GP1 between them positively identified as composed of neuronal Cx45.

In our study, the detailed localization and ultrastructure analysis were done using electron micrographs of rat longitudinal ultrathin sections of unmyelinated ON head region (Fig. 4A,B). We found that electrical synapses form axon–axon, astrocyte–astrocyte and axon–astrocyte connections (Fig. 4A,C); however, with immunogold labelling, only axon–axon synapses were positive for Cx45, showing that this neuronal connexin forms specifically axo‐axonal electrical synapses in the ON head (Fig. 4C). We were not able to obtain clear immunogold labelling of Cx36, most likely due to its lower expression in the ON. Detailed EM analysis of different gap junctions in ON head is presented on Fig. 5.

### Chemical blockage of ON gap junctions results in transient impairment of visual signal conduction observed in Visual Evoked Potentials

**Figure 6:**
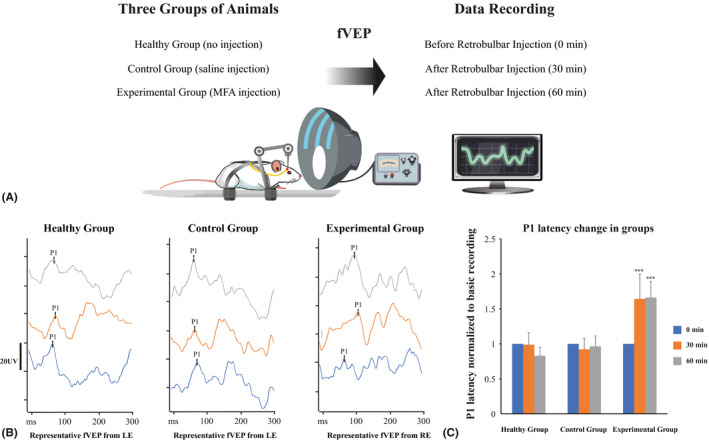
Functional testing of fVEP P1 wave latency in animals with and without chemical blocking of gap junctions. (A) schematic illustration of functional measurements protocol. Animals were divided into three groups consisting of five animals per group. Each measurement was repeated five times, and results were averaged. Basic recordings were done before any eye manipulations. Just after the basic recordings, animals (except the healthy group) received saline or MFA retrobulbar injections and consecutive recordings were performed. (B) representative fVEP recordings in an animal that received no injection, retrobulbar saline injection or retrobulbar MFA injection; each time‐point represents the average of five recordings in the same animal. In non‐treated animals and in animals that received retrobulbar saline injection, the P1 wave latency was unchanged in repeated recordings after 30 and 60 min in contrast to MFA‐injected animals, where GJs blocking resulted in elongation of P1 wave latency. (C) P1 wave latency change in animals within groups; the values of P1 latency were normalized to the basic recording from each group; ***p < 0.001, MFA = meclofenamic acid, ns = not significant.

To test the possible role of gap junctions in ON, we performed flash visual evoked potentials (fVEP) in rats to determine the impact of chemical blocking of electrical synapses on latency of the P1 wave, representing the time of the signal transduction from the retina to the occipital cortex (Fig. S1). Blocking the electrical synapses with retrobulbar injection of non‐selective GJ blocker, meclofenamic acid (MFA), which is an old generation non‐steroid anti‐inflammatory agent, resulted in significant elongation of P1 wave latency, which was spontaneously reversible over time. The measurements were performed at time 0, 30 min and 60 min after retrobulbar injection of MFA or saline (negative control). The average P1 latencies at time 0, 30 min and 60 min were 61.5 ± 8.3, 60.8 ± 10.5 and 51.1 ± 7.5 ms (p > 0.05, related samples Friedman's analysis); 64.8 ± 7.2, 59.8 ± 7.6 and 62.5 ± 10.1 ms (p > 0.05, related samples Friedman's analysis); 53.0 ± 7.4, 87.1 ± 8.4 and 88.1 ± 8.6 ms (p < 0.001, related samples Friedman's analysis) for non‐injected, saline‐injected and MFA‐injected groups, respectively (Fig. 6A–C). This transient blocking of GJs has probably dual mechanism, the metabolization of MFA as well as rapid exchange of blocked synapses with new ones.

## Discussion

Electrical synapses (gap junctions, GJs) are created by channel proteins connecting cytoplasm of neighbouring cells, thereby coordinating cell metabolic and electrical functions, including cell proliferation, differentiation, survival and apoptosis. Gap junctions (GJs) allow passage of small molecules, ions and secondary messengers between cells, synchronizing propagation of an action potential in excitable cell systems, including retinal neurons.

The presence of connexins expression (mostly connexins 36, 43 and 45) within the retina has already been described, (Völgyi et al. 2013a,b; Akopian et al. 2014, 2017; Bolte et al. 2016; Cowan et al. 2016; Kántor et al. 2016; Asteriti et al. 2017), and as elements of electrical synapses, connexins are involved in signal conduction and neuronal excitation control (Maxeiner 2005). Studies on connexin proteins in the ON are less numerous. The presence of GJ ion channels was confirmed in meningothelial cells (Cx36) (Zeleny et al. 2017) and GFAP‐positive ON astrocytes (Cx43) (Quigley 1977; May & Lütjen‐Drecoll 2002; Malone et al. 2007; Kerr et al. 2010; Cooper et al. 2016). Since Cx43 is the most studied GJs protein in the ON, experimental therapeutic approaches for optic neuropathies are also focused on this specific protein (Chen et al. 2013, 2015; O'Carroll et al. 2013).

The presence of neuronal‐specific connexins within the ON suggests their localization within RGCs axons, the only neuronal component of the ON. The combination of connexin proteins that comprise GJs in the central nervous system exhibits cell‐type specificity. Neuronal GJs are formed by Cx36 and 45 homo‐ or heteromers, astrocytes express GJs consisting of Cx30 and 43, and microglia, Cx36 and 43 (Orellana et al. 2013). The connexin expression panel in oligodendrocytes is still not fully known; however, it is unlikely that they express neuronal connexins (Abrams & Rash 2009). In recent papers, several types of axonal parallel side‐connections have been described, including gap junctions between hippocampal mossy fibres or axo‐myelinic synapses (Hamzei‐Sichani et al. 2007; Stys 2011). Visualization of GJ structure is especially difficult in nerve tissue because it requires atraumatic dissection of the nerve. Applying pressure on the axons may lead to ‘explosive‐damage’ of the cells, and the cell structure can easily be lost. Additionally, the short half‐life of connexins makes it crucial to plan experiments in a proper manner.

**Figure 7:**
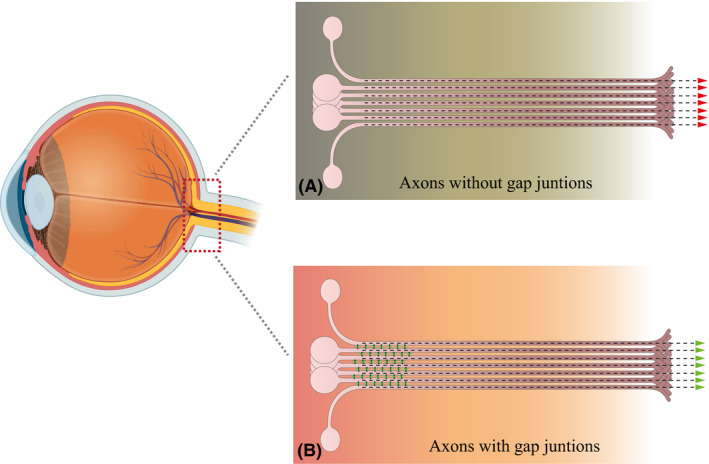
A novel model of optic nerve structure. Schematic illustration of conventional (A) and novel (B) models of optic nerve head structure. In the conventional model of the ON, the axons create independent, isolated channels allowing only for lengthwise current transduction. The new model of the ON considers the presence of interconnecting gap junctions between axons in the optic nerve head region that allow for lengthwise and crosswise propagation of impulses and decrease of the total ON resistance by creating syncytia that are connected in parallel, thus accelerating the current transduction.

Morphologically, the presence of axo‐axonal electrical synapses within unmyelinated region of the ON was undoubtable in our study, and therefore we aimed to determine the possible function of these connections. Neuronal GJs in the central nervous system have several functions, such as conduction of impulses, energy supply, cell volume regulation, propagation of intracellular calcium waves and homoeostasis, as well as neuroprotection (Hussain 2014). Here, we propose a new function of neuronal GJs. According to the ‘cable theory’, central nervous system tracts, including the ON, are composed of multiple parallel axons, each axonal membrane has its own resistance, and the entire resistance of the ON depends on single axon resistances (Tasaki & Matsumoto 2002; Akaishi 2017). This model would be possible only if each axon represents an isolated pathway, beginning in the RGC body and ending in the lateral geniculate nucleus, with no parallel splitting of the signal. This conventionally described arrangement does not favour signal conduction due to high resistances in the ON (Fig. 7A). Our finding gives new insight into the signal conduction process along the ON. Considering that each axonal membrane represents a specific resistor, axons, which express electric synapses, are side‐connected with each other, creating a system of parallel series of resistors. Since the structure of parallel series of resistors results, in principle, in lower resistance of the whole system, the presence of conductible electric synapses may play a role in the reduction of the total resistance of the ON, accelerating the transduction of impulses in the whole ON and allowing not only for lengthwise, but also crosswise conduction of the current (Fig. 7B). Moreover, the regulation of GJ conductivity itself could be a way of buffering, selecting and gating the visual signal that reaches brain visual centres. Similar arrangement of intercellular coupling of excitable cells has been described for cardiomyocytes, where uncoupling of cells is a determinant of slow conduction (Joyner et al. 1984). An additional aspect of the presence of GJs connecting neighbouring axons could be related to the ability of the current to induce changes in axonal membrane potential. Reducing membrane resistance makes the axolemmal potential easier to be initiated and, as a consequence, makes the impulse travel faster along the axon (Brzychczy & Poznanski 2014). Considering that ON axons originate from different types of RGC (belonging to ON‐ and OFF‐systems), the exact order and outcome of axonal coupling may be difficult to define and this aspect requires further investigation.

The newly described finding may contribute to understanding the complex mechanism of optic neuropathies, including glaucoma. Appearance of functional impairment that is observed in patients with optic neuropathies in early stages, before morphological changes (the thinning of the retinal nerve fibre layer) are present, could be related to impairment of axonal GJs, which is reversible initially. There are evidences that in early stages of optic neuropathies (e.g., in glaucoma); there is a discrepancy between morphological and functional changes in the ON, indicating that the ON must have some buffering system which allows protection of signal transmission despite progressive axonal damage (Malik et al. 2012). In early stages of glaucoma, morphological damage is more accelerated than functional impairment, and when the morphological damage becomes more advanced, the ratio of functional loss is proportionally greater than in early stages. Another aspect is the possibility of functional recovery of the ON, if the damage is very initial. Indeed, in glaucoma the visual field defects, if the stressor factors are eliminated (i.e., intraocular pressure is lowered), tend to reverse (Ventura & Porciatti 2005). Since some NSAIDs can reversibly block GJ function, exposure to high doses of NSAID can potentially affect ON function. There are reports, presenting temporary visual field and VEP defects after ibuprofen or MFA intake, which could have a pathogenic mechanism related to GJ blockage (Hamburger et al. 1984; Gamulescu et al. 2006; Sun et al. 2013).

The major finding in this paper is the description of the presence of previously unknown GJs (electrical synapses) between ON axons, which directly connect axons within bundles in the ON head. The presence of these GJs potentially accelerates signal transduction along the ON and allows modulation of the signal passage from the retina to the brain. By creating crosswise conduction within bundles of the ON, it could possibly allow bypass of local damage within axons. We hypothesize that density and conductivity of these synapses may be crucial with respect to the susceptibility of the ON to having different impairments develop into symptomatic pathologies. We showed that transient chemical blocking of ON electrical synapses slows down visual signal conduction. In the case of axonal structural or functional impairment, the signal could possibly be passed crosswise via GJs to the neighbouring axon; thus, the preservation of the syncytial structure of the ON can prevent the blockage of visual information propagation. This finding could have substantial implications for understanding of the pathogenesis of various optic neuropathies and identifies a new potential target for a therapeutic approach.

## Supporting information


**Fig. S1.** The method of VEP electrodes placement.
