# Supplementary figures and images for: Electrical synapses interconnecting axons revealed in the optic nerve head – a novel model of gap junctions’ involvement in optic nerve function
Source: Acta Ophthalmol. 2019 Oct 10;98(4):408–17. doi: 10.1111/aos.14272 (PMC7318195; doi:10.1111/aos.14272)

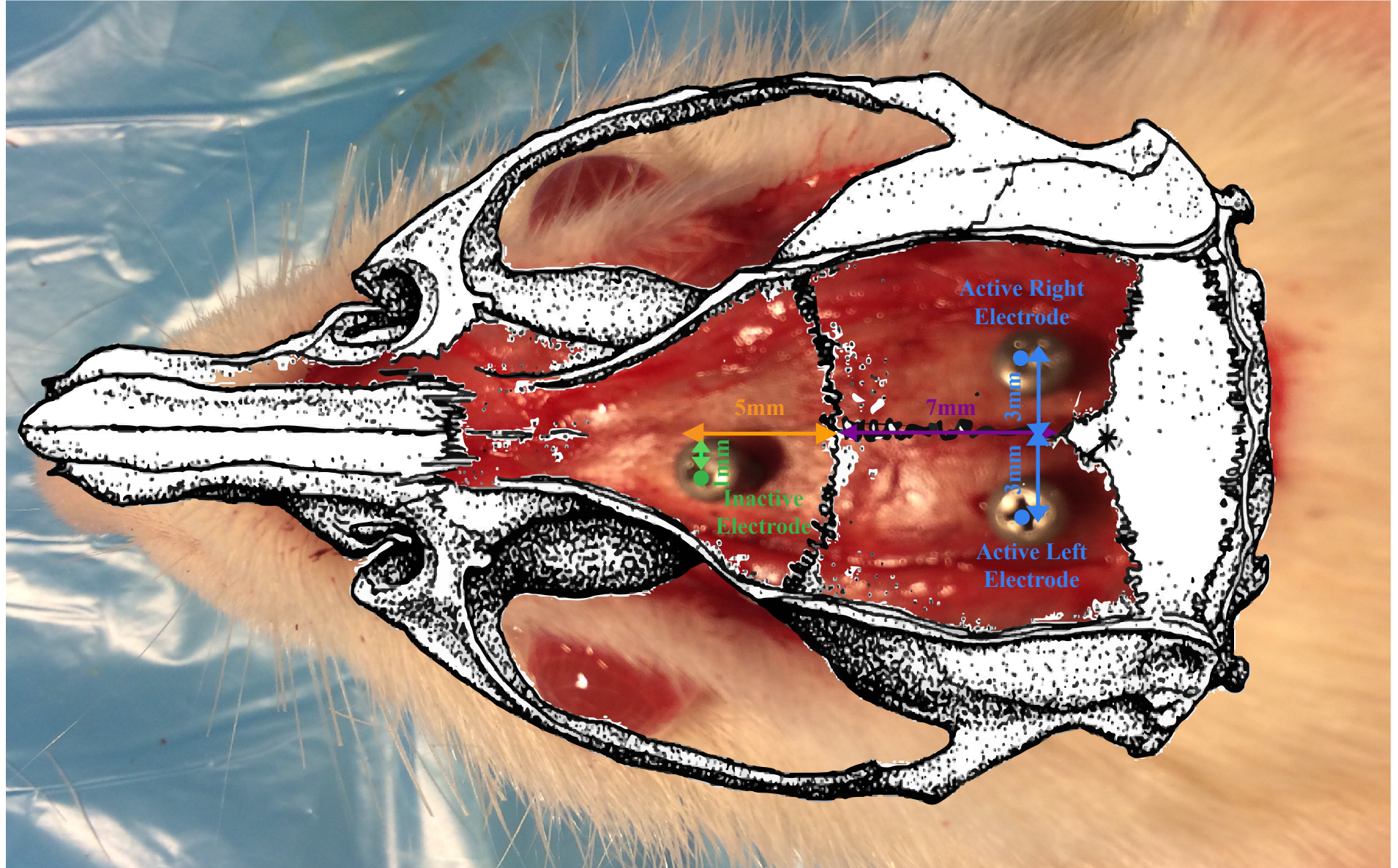

Supplement: Supplementary file 1 — Fig. S1. The method of VEP electrodes placement. [file AOS-98-408-s001.pdf]
